# Ride or Not to Ride: Does the Customer Deviate toward Ridesharing?

**DOI:** 10.3390/ijerph181910352

**Published:** 2021-10-01

**Authors:** Azra Shamim, Awais Ali Khan, Muhammad Ahsan Qureshi, Hamaad Rafique, Adnan Akhunzada

**Affiliations:** 1 College of Computing and Information Technology at Khulais, University of Jeddah, Jeddah 23218, Saudi Arabia; asmajeed@uj.edu.sa (A.S.); maqureshi@uj.edu.sa (M.A.Q.); 2 Department of Computer Science, COMSATS University Islamabad, Islamabad 44000, Pakistan; awaisrana436@gmail.com; 3 School of Computer Science and Technology, University of Science and Technology of China, Hefei 230026, China; hamaadrafique@mail.ustc.edu.cn; 4 Faculty of Computing and Informatics, University Malaysia Sabah, Kota Kinabalu 88400, Malaysia

**Keywords:** e-hailing applications (EHA), smart phones, technology acceptance model (TAM)

## Abstract

Traditional taxi services have now been transformed into e-hailing applications (EHA) such as Uber, Careem, Hailo, and Grab Car globally due to the proliferation of smartphone technology. On the one hand, these applications provide transport facilities. On the other hand, users are facing multiple issues in the adoption of EHAs. Despite problems, EHAs are still widely adopted globally. However, a sparse amount of research has been conducted related to EHAs, particular in regards to exploring the significant factors of intention behind using EHAs Therefore, there is a need to identify influencing factors that have a great impact on the adoption and acceptance of these applications. Hence, this research aims to present an empirical study on the factors influencing customers’ intentions towards EHAs. The Technology Acceptance Model (TAM) was extended with four external factors: perceived mobility value, effort expectancy, perceived locational accuracy, and perceived price. A questionnaire was developed for the measurement of these factors. A survey was conducted with 211 users of EHAs to collect data. Structural equation modeling (SEM) was used to analyze the collected data. The results of this study exposed that perceived usefulness, perceived price, and perceived ease of use affect behavior intention to use EHAs. Furthermore, perceived ease of use was impacted by effort expectancy, perceived locational accuracy, and perceived mobility. The findings of the study provide a foundation to develop new guidelines for such applications that will be beneficial for developers and designers of these applications.

## 1. Introduction

Transport services are a basic need for each field of life. Taxis or cabs are considered as a necessary component of mobility in the modern transportation system as compared to other kinds of public transports. The upside of taxicabs is that they incorporate quickness, protection, comfort, the absence of stopping charges, and 24 h services. Traditionally, people hail taxis on lanes, which is not much comfortable and less productive specifically in the case of rush hours or stormy days [1]. As the usage of technology is growing well ordered, transport services are transforming from having traditional to flexible application transportation advantages. The industry is adopting a new perspective for present-day transportation due to the advancement in mobile technology along with the increased use of mobile applications. Mobile applications are involved in numerous parts of our life, such as e-health, internet banking, e-learning, e-ticketing, e-shopping, e-libraries, etc., [2]. Likewise, people are utilizing smartphones for online booking applications for transportation e-hailing applications (EHA) at present. EHAs allow an individual to hail taxis through their cell phones. With the advancement of smartphone technology and the development of EHAs, it is now easy to hail a taxi through smartphones. Therefore, currently, people are utilizing EHAs for transportation reasons. Consequently, the taxi industry has been conventionally controlled in terms of fares [3]. In a small period, these applications have been adopted around the globe.

There are multiple EHAs for transportation such as Uber, Careem, Hailo, Grab Car, Yandex taxi, and Lyft [4]. EHAs were first presented in the UK in the year 2011 as the application named Hailo (latest version is known as “My Taxi”) for black taxicabs in London. In the last five years, EHAs are a widely used application in different cities of Pakistan and its demand is increasing day by day. In some big cities of Pakistan where traditional taxi services have a huge impact on the transportation system, EHAs makes transportation convenient and accessible. The advantages of EHAs include less searching and meeting time with the driver, easy communication between customers and drivers, and an easy payment method [1]. Conversely, there are some issues in the adoption of these applications. In Pakistan, EHAs completely changed the traditional taxi service. However, there are still multiple problems that people are facing in the adoption of such kinds of applications. Consequently, multiple issues occur while using EHAs and various factors affect the customer intention in the adoption of EHAs such as perceived price, usefulness, ease of use, effort expectancy, trust, and locational accuracy. Therefore, users demand more sophisticated and user-friendly EHAs. A variety of factors may contribute towards the adoption and usage of EHAs. However, limited research has been conducted related to EHAs that highlight vital factors of intention to use EHAs.

The transportation industry is a basic industry and currently a significant portion of the revenue of this industry has been generated by EHAs [5,6]. In the context of Pakistan, the revenue generated by EHAs in 2020 was US$265 m. Moreover, the annual expected growth rate from this revenue is 18.4%, which results in a $440 m market volume by 2023. The user penetration rate of EHAs was 5.3% in 2020 and a 7.1% user penetration rate is expected by 2023 [7]. The expected increase in user penetration rate and market volume shows the significance of EHAs. Therefore, the aim of the current study is the acceptance of the EHA “Careem” in an empirical manner. The Careem application is popular in many countries around the globe and it is on the list of the top ten applications in Pakistan. However, the adoption factors may differ in each region. Therefore, the goal of this paper is to investigate the features influencing the user’s intention to the Careem application in Pakistan. Moreover, the research explores the factors that resulted in low adoption and usage of the Careem application by its users in Pakistan. A wealth of evidence was reported in the literature which uses the technology acceptance model (TAM) as a base model to prove the acceptance of technology by society [8,9,10]. Hence, the proposed study extended TAM by integrating external factors (locational accuracy, perceived mobility, effort expectancy, and perceived price) to investigate the usage behavior of the Careem application in the context of Pakistan. A questionnaire survey was developed and conducted to get responses from the users of the Careem application. Authors [8,9,10,11] in their study adopted TAM to measure the acceptability of the systems and for verifying the hypothesis they have adopted the structural equation model (SEM), as it is the best model to measure the influence of the factors on the dependent variables. Therefore, the proposed study will use structural equation modeling (SEM) to deeply understand the influential factors of the Careem application in the Pakistani context.

This paper comprises six sections. Section 2 discusses a review of existing literature. The proposed research prototypical and theory are described in Section 3. The research method adopted to carry out the work is explained in Section 4. Section 5 comprises results and discussion. The last section concludes this research work.

## 2. Literature Review

### 2.1. Information System Acceptance Models

Understanding the acceptance and rejection of new technology by its users became the key line of research in the information system scope. Therefore, different theories and models were proposed to investigate the aspects influencing intention to use information systems [4], including the theory of planned behavior (TPB), theory of reasoned action (TRA), and TAM. TRA, projected by [12], is used to determine the relationship between behavioral intention and attitude. TPB was proposed by [13] and it is the extended form of TRA by the addition of the variable “perceived behavioral control”. TAM is the extension of TRA and was developed by [14]. According to the TAM, the behavior of individuals is identified by their behavior intention (BI). TAM has two essential factors: perceived ease of use (PEOU) and perceived usefulness (PU). Table 1 shows the factors included in TRA, TBP, and TAM. TAM was widely used to predict user’s intention toward new technology and to identify features influencing the adoption of technology in numerous settings/domains (online banking, digital library, mobile payment, hospital system, EHAs). TAM was widely used to explore users’ behavior towards new technology related to transportation.

### 2.2. Related Work

Existing studies utilized different models to identify influencing factors. Some of the studies extended these models according to domain and context. The study [2] pays to the progress of a new theoretic context by combining the TAM and accounting theory while targeting the mobile ticketing application. SEM was used to observe the hypotheses based on the planned theoretical framework. Similarly, various other studies used SEM with TAM to verify the acceptance of EHAs among users [10,11]. In [15], TAM, diffusion of innovation model, and UTAUT model were utilized to propose a combined model called integrated model on mobile payment acceptance, and this model is considered suitable for mobile payment applications in community transportation. The study [16] suggested that an information system continuance model should examine the effect of satisfaction and PU on users’ post-adoption behavior. The author merged perceived usefulness into ECT (expectation confirmation theory), to explain customers’ information system continuance intentions. The study [17] examines the intent of mobile taxi booking application’s users. For this purpose, three models (ECT, TAM, and the cognitive model) have been used. The model used in the existing literature is shown in Table 2. The influencing factors extracted from literature in the domain of transportation services are presented in Table 3. A summary of existing studies related to the transportation industry in different contexts is presented in Table 4.

## 3. Proposed Model and Hypotheses

Prototypical research is projected in this study in the setting of EHAs founded on the findings of the literature review, where Pakistan is chosen as the study context. Pakistan is ranked amongst the topmost five Asian states in which the usage of smartphones and the internet is increasing day by day [50]. The information technology sector in Pakistan is growing rapidly. However, the acceptance of new technologies was found to be low due to less strong mobility factor in Pakistan that results in less user satisfaction and ease of use [51].

**Figure 1:**
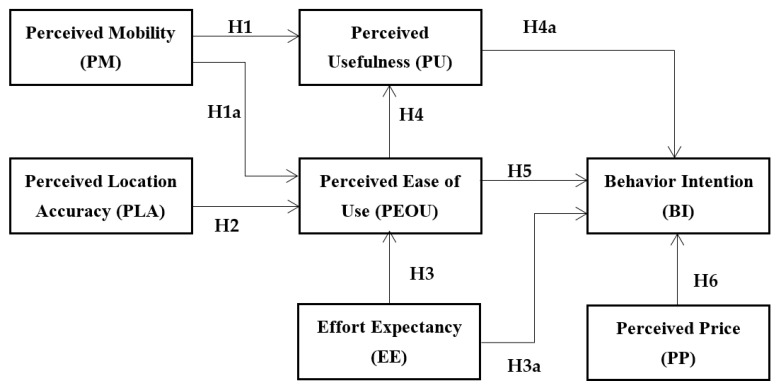
Proposed Research Model.

To achieve the aim of this work, the technology acceptance model (TAM) is adapted in the study due to three reasons: (i) it is widely used by different researchers in various domains; (ii) it assists in understanding the users’ viewpoint; and (iii) it allows exploring the impact of external variables on a dependent variable. Based on the findings of the literature review, external factors (namely, perceived mobility, perceived locational accuracy, effort expectancy, and the perceived price) were added to TAM. The reason for adding perceived mobility is of noteworthy importance. It is due to the fact that the stability of the internet connectivity on the move in Pakistan is not strong enough that it will help the customer or driver to reach an accurate position for each other. Therefore, due to bad connectivity, extra efforts were needed to access the ride which might increase the price of the ride. Hence, it is important to understand the influence of the external factors toward the acceptance and usage of EHAs while keeping the low price of a ride with accurate location on the move. Our proposed model is presented in Figure 1.

### 3.1. Independent Variable: Perceived Mobility (PM)

PM is defined as ‘the degree to which a person trusts that using mobile apps would improve their capability to cope with tasks and activities while on the move.” [52]. According to [53], PM refers to the consumers’ feelings and awareness of the portable value of services offered by smartphones. The mobility allows users to access the resources at any time and any place. The advantages of PM include immediacy, convenience, and expediency as discussed in [54]. The primary benefits of mobile services are the mobility of devices [54,55,56]. PM is one of the strong and indispensable representatives of mobile technology acceptance and adoption [28,52,57]. The existing literature highlighted a significant impact of PM on adoption; the increase in mobility will increase the time spent on mobile service [28]. Currently, users are traveling long distances for holidays, shopping, school, and work as compared to many years ago. Therefore, they need more sophisticated applications to perform a variety of tasks while they are on go [58]. PM facilitates users to access their smartphones anywhere and anytime [59]. The mobility makes users free from temporal and spatial constraints by accessing resources at any time and any place [60]. As a result, users can use their mobile phones to perform various activities while traveling [61].

Similarly, [62] revealed that users can purchase items anytime and anywhere using their smartphones. PM was depicted as a strong predictor of BI in many past studies conducted in diverse domains such as online purchases, online banking, mobile applications [52], mobile library applications [60], e-book devices [53], ride-sharing [58], and location sharing [59]. Empirical research has reported the significant impact of mobility on PEOU and PU [54,56,60,63]. Additionally, [54] revealed a strong link between PU and PEOU. The user penetration rate of E-hailing is increasing day by day in Pakistan. One of the reasons behind this might be the increased use of smartphones in Pakistan with a penetration rate of 35.9% (according to the latest statistics of December 2019 reported by the Pakistan Telecommunication Authority). Further, Pakistan is ranked among the top five Asian countries according to the usage of smartphones and the internet (www.unctad.org, accessed on 20 January 2021). The smartphone and the internet provide mobility values to users. Therefore, the researcher integrated PM with TAM in the e-hailing domain. Hence, the following hypotheses can be stated:

**Hypotheses** **1** **(H1).** *PM has a positive influence on PU in EHAs.*

**Hypotheses** **1a** **(H1a).** *PM has a positive influence on PEOU in EHAs.*

### 3.2. Independent Variable: Perceived Locational Accuracy (PLA) 

Perceived locational accuracy is defined as the degree to which users of EHAs are becoming aware of their accurate current locations. PLA is described as how much users of EHAs are aware to their precise current location. In the scenario of navigational apps, the application should present correct guidelines that take the user to their destination efficiently due to real-time usage [22]. Therefore, the higher viability and locational accuracy of EHAs will result in more use of the application. [22] Showed an impact of perceived location accuracy on PU. Providing location information was one of the most important features of EHAs. Sometimes the global positioning system faces problems of accuracy due to a variety of reasons spanning from atmospheric to terrestrial factors. The atmospheric reasons might be changes in pressure, temperature, and humidity that lead to accuracy errors. On the other hand, signal interference along with weak satellite signals are the reasons for less accuracy. Further, without a clear and strong signal, it is difficult to get the exact location. However, in EHAs, location accuracy plays an important role. The current study proposes the following hypotheses based on this rationale:

**Hypotheses** **2** **(H2).** *PLA of EHAs has a positive influence on PEOU.*

### 3.3. Independent Variable: Effort Expectancy (EE) 

Effort expectancy is defined as the effort needed to complete a task using a given IT/IS [64]. EE is the second core component in the UTAUT model. In the context of m-taxis, EE is the expectation of prospective consumers that the application is free of effort and represents understandability [51]. In the literature, effort expectancy had a significant impact on the adoption and behavior intention to use EHAs [3,44]. In the context of mobile technology, BI was found to be more likely when the technology is less complicated [65]. Ref [66] highlighted that if the time required and effort required to learn a technology is less, then it is more likely to be accepted by its users. [67] reported a relationship between the complexity of tasks and the confidence of users. This relationship consequently influences perceived difficulty in using the system by its users. A positive association between EE and BI has been identified in the context of mobile technologies, particularly in mobile payment [68] and mobile marketing [69]. In the context of a mobile tourism application, a positive link was found between EE and BI [70]. Similarly, the relationship between EE and BI was confirmed in the domain of automated road transport systems [18]. If users required less mental effort to use the EHA then they are more likely to adopt these applications. According to (www.statista.com, accessed on 10 January 2021) the penetration rate of smartphones in Pakistan for 2020 was estimated to be 51%, showing that people are adopting smartphones to perform a variety of tasks using mobile applications. The Careem application is in the top ten most popular applications of Pakistan (www.mediabites.com.pk, accessed on 23 March 2021) (www.researchsnipers.com, accessed on 23 March 2021). This ranking, exhibiting that a lot of mobile users are using it, might be due to the fact that less effort is required to use the application. Therefore, the following hypotheses are proposed:

**Hypotheses** **3** **(H3).** *EE has a positive influence on BI in EHAs.*

**Hypotheses** **3a** **(H3a).** *EE has a positive influence on PEOU in EHAs.*

### 3.4. Independent Variable: Perceived Ease of Use (PEOU)

PEOU is the degree to which an individual perceived the asserted effort in using the application [71]. If the application is easy to use, then it will positively influence the intention to use the EHA. The impact of PEOU on PU was reported in the ride-sharing scenario by [29]. Another study confirmed the same relationship in the adoption of EHAs in the context of Brazil [30]. The following hypotheses are proposed:

**Hypotheses** **4** **(H4).** *PEOU of EHAs has a positive influence on PU.*

**Hypotheses** **4** **(H4a).** *PEOU of EHAs has a positive influence on BI.*

### 3.5. Independent Variable: Perceived Usefulness (PU)

PU is defined as the degree to which a person trusts that using a specific system would improve their job performance [14]. In the literature, perceived usefulness had a significant impact on the adoption of ride-sharing applications [29]. The relationship between PU and BI has been confirmed in another study that focused on drivers’ intentions to use car navigation systems [22]. However, the impact of PU on BI was not found in [29,30]. This research study hypothesis:

**Hypotheses** **5** **(H5).** *PU has a positive influence on BI of EHAs.*

### 3.6. Independent Variable: Perceived Price (PP)

PP is defined as consumers’ cognitive trade-off between the perceived benefits of the applications and the monetary cost of using them [3]. Perceived price is described as the degree to which the users are getting the benefits of the application at a lower price [3]. The lower the price value the higher will be the intention to use EHA. Generally, the taxi charges are higher as compared to the e-hailing application (local cab services) [58]. Further, the rates of Uber are less than taxi even in peak hours [72]. The price offered by EHAs is a decisive parameter to adopt EHAs. Therefore, the following hypothesis is proposed to explore the impact of price on the usage of EHAs:

**Hypotheses** **6** **(H6).** *PP has a positive influence on the BI of EHAs.*

### 3.7. Dependent Variable: Behavioral Intention (BI)

System acceptance is the interpreter of BI [14]. User behavior is defined by usage intentions in technology acceptance studies. A high correlation is reported between behavior and intention [73]. However, the intention is the direct antecedent of behavior [74]. Literature reported the significant direct association of PEOU and PU with usage intention; therefore, BI is selected as a dependent variable in this work.

## 4. Research Methodology

### 4.1. Questionnaire Development

To confirm the reliability and validity of the proposed model, a questionnaire was developed in which each measurement item was adapted from existing studies. The questionnaire contains two parts: the demographic part and questions related to selected factors. The first part asked questions about age and gender. In the second part, six factors were discussed, and their influence was measured using a five-point Likert scale that ranges from strongly disagree to strongly agree. The items of the factors (PU and PEOU [75], PM [56], EE [76], PLA [22], PP [77], and BI [14]) were adapted from related studies. There were five items for PU, four items for PEOU, PLA, PP, and EE, and three items associated with BI. The selected factors influence the adoption of EHAs. Behavior intention was the dependent variable while perceived ease of use, perceived mobility value, locational accuracy, perceived usefulness, effort expectancy, and perceived price were the independent variables. The questionnaire is attached in Appendix A.

### 4.2. Data Collection

A questionnaire survey (quantitative approach) was conducted in the context of Pakistan, where EHAs are widely used by the people. Data were collected from the users of EHAs in different cities of Pakistan where EHA services are available. Respondents were invited to fill in the questionnaire by providing their agreement level. The questionnaire was distributed online to different users of EHAs. A total of 211 respondents filled the questionnaire in which 102 (48.3%) were male and 109 (51.7%) were females. Of the 211 respondents, 92.4% were using the EHA and 7.6% were not using an EHA. Respondents’ details are shown in Table 5 The majority of the respondents were students as it can be seen from the respondents’ profile that about 51.6% of responses were from the age group of 26 to 30 years of age followed by the age group of 20 to 25 years of age (i.e., 19.9%). Before collecting data, only those students were contacted who is a regular user of the e-hailing application. Therefore, demographic information regarding the usage frequency of EHAs or usage of mobile phone were eliminated from the questionnaire.

### 4.3. Data Analysis

Data analysis was conducted by using two software: statistical package for social science (SPSSv20) and analysis of moment structure (AMOS v20). Data coding and cleaning was performed in SPSS. It was also used for factor analysis that consists of exploratory factor analysis (EFA) and confirmatory factor analysis (CFA). The testing and validation of the proposed hypothesis and constructs was performed using SEM in CFA with the help of the AMOS tool. SEM consists of a measurement model (MM) and the structural model (SM). The MM tests the reliability, validity, and goodness of fit indices while the SM tests the proposed hypothesis. The measurement and structural models were performed by AMOS.

### 4.4. Data Screening

Data screening is the process to handle redundancies and mistakes from the data to make it clear at the initial stage before performing further analysis. The data were coded in SPSS. Data screening was accomplished to check the reliability and validity of data before data analysis. First, the randomness of missing data was identified by applying Little’s Chi-square test, and the obtained *p*-value of 0.343 (which is greater than 0.05) shows that data were missing completely at random. Consequently, the regression imputation technique was applied to fill in the missing values. Unengaged responses and the same response for all variables were removed. Skewness and Kurtosis values were used to check the normality of the data. The value of skewness ranged at ±1 and the value of Kurtosis ranged at ±3. The positive value of Kurtosis means that there was less variation in the data. The respondent’s responses to the variables were in a very similar way. The negative value of the Kurtosis means that the respondents answered differently and there was not a central tendency towards the median. In the end, an outlier was detected from the data at both univariate and multivariate levels. Outliers are the values that are fluctuating evocatively from the mean values. Therefore, outliers at the univariate level were measured with the help of Z-score and at the multivariate level with the help of the Mahalanobis D^2^ test. So, based on the statistical rules, if the value of D^2^/DF is greater than 3–4 then it is an outlier at the multivariate level, and if the value is 3 SD away from the mean then it is an outlier at the univariate level. The obtained results show that the values of outliers were within the defined range for both types with the insignificant nature of the outlier. Hence, outliers were kept in the study to maintain the generalizability of the study.

## 5. Results and Discussion

The large pool of data needs to be transformed into minor components to get a clear explanation of the data and for this reason, factor analysis (FA) was performed. FA is further divided into two steps: EFA and CFA. EFA was conducted by using SPSS for the purification of items. The Kaiser–Mayer–Olkin test and Bartlett’s test of sphericity were performed for EFA to measure the sampling adequacy. The obtained value of KMO was 0.825 whereas the *p*-value of Bartlett’s test was <0.001. Principal component analysis and Varimax rotation with Kaiser normalization was used for the extraction and rotation of the items, respectively. Factor loading values above 0.6 were considered eligible for further analysis. Secondly, extraction of factors was performed with an eigenvalue greater than one with a total of seven extracted factors with a variance of about 67%. After EFA, these factors were checked by performing CFA using SEM with the help of AMOS. It was conducted to check the reliability and validity of the constructs by hypothesis testing. SEM was used due to the adequacy toward the estimation of the structured relationship among the integrated constructs with multiple items in multivariate situations. SEM was used to analyze the relationships at the multivariate level among latent and measured variables. It was performed with the help of recommended two steps approached as used in [78]. The approach was divided into structural and measurement models (SM, MM). The output generated from SEM analysis was in the form of the goodness of fit indices. Those fit indices will be used to identify the fitness of data against the proposed hypothesis in SM as well as in MM.

### 5.1. Measurement Model

The construct’s reliability and validity check were the main steps toward the evaluation of the hypothesis. Therefore, it was done by measuring Cronbach’s alpha, composite reliability (CR), and average variance extraction (AVE) of the constructs and items. The reliability of the items depends on the achieved results in such a way that they are meeting the threshold values s. The results of the measurement model are presented in Table 6. The values for factor loading are greater than the threshold value [79], indicating the reliability of constructs. Table 6 revealed that the composite reliability of each factor is above 0.7 (higher than the threshold value of 0.7 recommended [80]), except for PEOU. Moreover, the values of AVE are greater than 0.5 (the threshold value recommended by [79]). Furthermore, the square root of obtained AVE values was taken to measure the discriminant validity as shown in Table 7. The results of the fit indices are presented in Table 8 and indicate that the MM satisfactorily fitted the data. Also, the value of Cronbach’s alpha was above 0.70 (which is the acceptable value).

### 5.2. Structure Model

This is the phase that helps in the verification of the proposed hypothesis. It was performed in AMOS with the help of the coefficient of determination (R^2^) and path coefficient. The strength of the proposed hypothesis was checked and verified with the path coefficient and construct supremacy was determined with R^2^. The statistical values achieved during the analysis for both R^2^ and the path coefficient support the data for the hypothesized model [78]. It helps in supporting the proposed relationships with the help of fit indices just as in the measurement model. The results for fit indices for both MM and SM are presented in Table 8 along with the threshold values.

**Figure 2:**
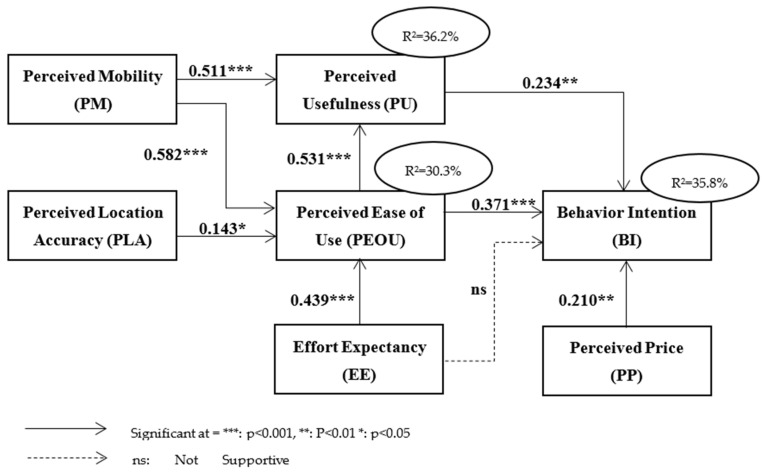
Pictorial representation of results.

Figure 2 and Table 9 represent the results of SM. All of the accepted paths have values within the range of 0.001, 0.01, or 0.05. The obtained results are representing the noteworthy acceptance of all the integrated factors of the study toward the usage intention of e-hailing applications for daily use. The structure model explains the relationships of the hypothesis. The results of SM show that eight hypotheses (H1, H1a, H2, H3, H4, H4a, H5, and H6) are supported as shown in Table 8. However, the H3a hypothesis is rejected.

**H1: PM** **→PU, H1a: PM** **→PEOU**

The relationship between PM and PU is significant (β = 0.511 *** and CR = 6.615 whereas *p* = 0.001), as the *p*-value is less than the threshold value (*p* ≤ 0.05) and the critical ratio is higher than the threshold value (1.96). Therefore, H1 is supported by these values indicating a significant influence of PM on PU. Similarly, the relationship between PM and PEOU was found significant (β = 0.582 *** and CR = 5.214 whereas *p* = 0.001), Thus, H1a is also accepted and an impact of PM on PEOU was exhibited. These results indicate that PM affects both core components of TAM (PU and PEOU). The results are in line with previous studies [54,56,60,63].

**H2: PLA** **→ PEOU**

Hypothesis H2 was expected to have a strong influence on the dependent variable. However, it was not proven right and the statistical results revealed a weak significant relationship between PLA and PEOU based on the values (β = 0.143 * and CR = 2.779 whereas *p* = 0.03). Therefore, it can be defined that, PLA has a weak influence on behavior intention through PEOU. This result also follows the previous study [22].

**H3: EE** **→PEOU, H3a: EE** **→BI**

The EE was found to be influential for PEOU (β = 0.439 *** and CR = 4.939 whereas *p* = 0.001). However, the relation of EE with BI (β = −0.52 and CR = −0.582 whereas *p* = 0.560) was not established. Therefore, H3 is supported by the results and H3a is not. These results describe an impact of EE on PEOU while the impact of EE on BI was rejected. The association of EE and BI was also supported by literature in [58,59,60,61,62,63,64,65,66,67,68,69,70,81]. The reason behind the non-significant influence of EE on BI might be that the digital persons were native to the digital technology as they were born and grew up in the latest technologies. Therefore, they are much more familiar with the latest cutting-edge technologies. Thus, the uber technology that requires the use of computers or the internet might be considered as an ordinary thing for them. Hence, the results are in parallel to the exiting study [81].

**H4: PEOU** **→PU, H4a: PEOU** **→BI**

The hypothesis H4 and H4a were accepted as the relationships between PEOU and PU (β = 0.531 *** and CR = 7.428 whereas *p* = 0.001), and PEOU and BI (β = 0.371 *** and CR = 4.987 whereas *p* = 0.001) are confirmed by β value, critical ratio and *p*-value. The acceptance of H4 and H4a highlights the association of PEOU with PU and BI. These results are in line with the results reported by studies [29,30].

**H5: PU** **→BI**

The relationship between the path PU and BI is significant (β = 0.234 ** and CR = 2.043 whereas *p* = 0.008). Therefore, H5 said that PU has a significant influence on BI. A similar result was reported by [22].

**H6: PP** **→BI**

The impact of PP on BI is significant (β = 0.210 ** and CR = 2.878 whereas *p* = 0.004. Therefore, H6 said that PP has a significant influence on BI which is acceptable and H6 is supported by these values.

This study identified PM, PLA, EE, and PP as key determining factors of users’ perception of EHAs. These factors were extracted by conducting a thorough literature review. Then, a conceptual model was introduced that extended well-known model TAM with external factors to investigate users’ behavioral intention towards EHAs. The results of this study statistically support all hypotheses of basic TAM. Additionally, this study presented the impact of PEOU and PU on the perception of users towards the e-hailing application in the Pakistani context. PM (β-value = 0.582) has strongest influence on PEOU followed by EE (β-value = 0.339). Moreover, the lowest impact of PLA (β-value = 0.143) on PEOU was also discovered. These results indicate that PM is a strong determinant of PEOU. Moreover, it can be concluded that the PEOU of e-hailing can be improved by enhanced PM based on the significant association of PM and PEOU. On contrary, PM had less impact on PU (β-value = 0.311) as compared to PEOU (β-value = 0.531). The extended model described that PM is a vital factor in the exploration of users’ behavior towards e-hailing application acceptance. Further, the contribution of PP in the exploration of users’ attitudes and acceptance toward EHA is highlighted by the extended model. Furthermore, the association of PM with PEOU and PU is offered by the extended model. The findings of this study provide significant contributions to prior TAM studies along with literature regarding EHA. Moreover, the outcomes of this work are an addition to existing research by emphasizing the relationship of PM, PP, PLA, and EE with constructs of TAM in the Pakistani context.

## 6. Contributions and Implications of the Study

### 6.1. Theoretical Contributions

This study proposed a model for the acceptance of new technology in the ridesharing context. This study extended the TAM model by adding external factors from the relevant literature to measure the influence of the riders toward shared services. The achieved results show the effectiveness toward strengthening the concept of acceptance of e-hailing in the context of Pakistan. Therefore, the proposed model can be used as a base model toward measuring the acceptance of technology in various other contexts. Secondly, the study reports the influencing factors of the e-hailing application adoption by proposing and validating a research model to understand users’ behavior concerning the e-hailing application. Thirdly, the relationship between the factors described in this work extend the literature of information systems, especially in the transportation sector. The outcomes of this research can be applied to investigate the acceptance of new technologies and applications. Also, the findings of this study can be utilized to examine users’ behavior towards e-hailing applications in a different context. Additionally, the results of this study may help designers of these apply to design and develop applications that will have high acceptance from users.

### 6.2. Practical Implications

The findings from the empirical study present several effective practical implications to policymakers, users, and developers of EHAs to promote the use of these applications. The study stated vital factors in the acceptance of EHAs by users. These factors should be handled properly during the development of the new EHAs to increase the acceptance of EHAs. One of the crucial factors is PM, which has an impact on both core components of TAM. Therefore, it requires considerable attention. As the findings suggested a direct impact of PP on behavioral intention, these applications must provide a cheap price for its customers. Otherwise, customers may prefer the traditional taxi model. The developers of these applications are recommended to provide adequate importance to PLA, EE, and PM as these factors influence ease of use. If an application is not easy to use then it has low usage and adoption problems. The developers may enhance the usage of these applications by focusing on the PEOU factor. The benefits of applications should be brought to the notice of its customers to increase the adoption of these applications. This can be achieved by the positive impact of PM, PLA, EE, PP, PEOU, and PU. Additionally, the barriers to the popularity of these applications are highlighted. Existing EHAs can be improved by targeting the barriers to get high acceptance from users. The outcome of this study can be used as a contribution in the development of new guidelines for EHAs in such a way that it will help developers or market operators in understanding the willingness and intentions of users toward their updated services. In addition, these findings can be used as a base for the new startups as it will help them in improving their services and performance to improve their standard in this high competition environment.

## 7. Conclusions

As the use of technology is increasing day by day, the use of EHAs is also increasing in Pakistan. Everyone needs quicker and convenient transport. This study helps in understanding the usage intention behind users of EHAs. The behavior of users can be affected by many factors, so the conceptual model in this study helps in understanding the usage intention of EHA. The findings of this study helped in proposing some guidelines for the developers and designers while developing these applications. The results show that PM, PLA, PU, PEOU, EE, and PP are factors that are influencing the adoption of EHAs and therefore developers need to consider these factors while developing EHAs. This work has some limitations. First, the limitation of the study is its generalizability. The results of this study cannot be generalized to other contexts as the IT growth and infrastructure of Pakistan is different from other countries. The second limitation is that the data is collected mostly from students (young and educated people) who are more technologically aware. Therefore, the results for the older adult group may be different. Future research may be conducted further to explore the effect of other external factors on EHAs. Another future direction can be the investigation of the usage behavior of e-healing applications among older adults and less educated people.

## Figures and Tables

**Table 1:** Factors of TRA, TPB, and TAM.

Factors	Models		
	**TRA**	**TPB**	**TAM**
BI	✓	✓	✓
Attitude	✓	✓	✗
SN	✓	✓	✗
PBI	✗	✓	✗
PEOU	✗	✗	✓
PU	✗	✗	✓

**Table 2:** Models used In Literature.

SR	Method/Model	Study
1	TAM	[2]
2	TAM, DOI, UTAUT model.	[15]
3	TAM, ECT	[16]
4	TAM, TCT	[17]
5	UTAUT	[18]
6	TAM, UTAUT	[19]
7	UTAUT2, DOI	[3]
8	TAM, UTAUT	[20]
9	TAM	[21]
10	TAM, TPB	[4]
11	TAM	[22]
12	ACSI model, PSI model	[23]
13	TPB	[24]
14	IDT, TAM, TRA and TPB	[25]
15	TAM	[26]
16	TAM	[27]
17	UTAUT	[28]
18	TAM	[29]
19	DOI	[30]
20	DOI, TAM	[31]
21	TAM, ECM	[32]

**Table 3:** Influencing factors.

Sr. No	Influencing Factors	Studies
1	Perceived ease of use	[7,15,17,21,25,26,29,30,31,32,33,34]
2	Perceived usefulness	[2,7,15,16,17,21,22,25,26,29,30,32,33]
3	Perceived safety	[7,20,23,27,35]
4	Perceived price	[3,7,23,27,36,37]
5	Perceived convenience	[23,27]
6	Perceived accessibility	[27]
7	Perceived risk	[2,3,17,19,29,37,38,39,40]
8	Compatibility	[3,15,25,30,38,41,42]
9	Security	[15,23,31]
10	Perceived locational accuracy	[22,23,43]
11	Time benefit	[36]
12	Effort expectancy	[3,18,19,20,28,44]
13	Social influence	[3,18,19,20,24,28,36,31,41]
14	Privacy concern	[3,19,38]
15	Facilitating conditions	[3,19,20,23]
16	Complexity	[25,30,31,34]
17	Relative advantage	[25,30,31,41,45]
18	Trust	[19,25,30,36,38,39,44,46]
19	Anxiety	[20,36,28,47]
20	Personal innovativeness	[2,3,15,26,40,46]
21	Behavioral intention	[2,15,16,18,20,21,22,24,26,28,29,37]
22	Attitude towards using	[17,20,22,24,26,33,36,41,43]
23	Satisfaction	[16,17,22,25,32,48]
24	Confirmation	[16,17,32]
25	Subjective norm	[24,25,30,33,37]
26	Performance expectancy	[3,18,19,20,28,34,44,49]
27	Self-efficacy	[20,32]
28	Service Quality	[38,48]

**Table 4:** Literature summary.

Reference	Domain	Context	Findings
[16]	Online Travel Services	China	User satisfaction and usefulness impact the user intention toward the use of online travel services
[17]	Mobile Taxi Application	Malaysia	Attitude, PU, and satisfaction are considered important factors in the intention to use mobile taxi applications.
[30]	E-Hailing Applications	Brazil	Perceived usefulness has positively influenced user satisfaction. User trust also influences the intention to use EHA.
[19]	Location-based Services	China	A strong positive relationship among PEOU, PU, and trust on intentions to use was found.
[18]	Automated Road Transport Systems	France	Effort expectancy, social influence, and performance expectancy influence the behavior intention to use the system.
[33]	Ridesharing Applications	Vietnam	PU and PEOU are positively related to attitude.
[31]	E-Hailing Applications	Thailand	Relative advantage and PEOU influence the intention to use EHA.
[2]	Mobile Ticketing	Taiwan	Perceived risk, PU, and PEOU affect the intention to use Mobile ticketing applications.
[29]	Ridesharing service	China	Display quality, service, locational accuracy, perceived processing speed, and customer satisfaction are the influencing factors toward the use of ride-sharing services.

**Table 5:** Respondent profile.

Demographic Factors	Categories	Frequency	Percentage
Gender	Male	102	48.3
	Female	109	51.7
Age	Less then 20 years	21	10
	20–25 years	42	19.9
	26–30 years	109	51.6
	31–35 years	31	14.6
	Above 35 years	8	3.8

**Table 6:** Standardized item loadings, AVE, CR, and alpha values.

Constructs	Items	Factor Loading	CR	AVE	Cronbach’s Alpha
Behavioral Intention (BI)	BI1	0.749	0.781	0.737	0.747
	BI2	0.738			
	BI3	0.724			
Perceived Price (PP)	PP1	0.774	0.760	0.716	0.717
	PP2	0.742			
	PP3	0.632			
Perceived Locational Accuracy (PLA)	PLA1	0.761	0.765	0.721	0.728
	PLA2	0.738			
	PLA3	0.665			
Perceived Ease of Use (PEOU)	PEOU1	0.743	0.653	0.613	0.717
	PEOU2	0.719			
	PEOU3	0.677			
Perceived Mobility Value (PM)	PM1	0.817	0.770	0.725	0.703
	PM2	0.718			
	PM3	0.641			
Effort Expectancy (EE)	EE1	0.747	0.763	0.720	0.701
	EE2	0.711			
	EE3	0.703			
Perceived Usefulness (PU)	PU1	0.799	0.723	0.676	0.706
	PU2	0.738			
	PU3	0.693			

**Table 7:** Discriminant Validity.

Correlations Squared	BI	PP	PLA	PEOU	PM	EE	PU
BI	**0.74**						
PP	0.36	**0.71**					
PLA	0.23	0.44	**0.72**				
PEOU	0.27	0.28	0.49	**0.61**			
PM	0.39	0.32	0.38	0.46	**0.72**		
EE	0.32	0.46	0.34	0.31	0.30	**0.72**	
PU	0.44	0.33	0.21	0.25	0.27	0.33	**0.67**

Note: Diagonal (i.e., bold) values are AVE and off-diagonal are inter-construct correlations.

**Table 8:** Summary of fit indices.

Absolute Fit Measure				Parsimonious Fit Measure		Incremental Fit Measure	
	*p*-value	RMSEA	GFI	CMIN	CMIN/DF	CFI	TLI
Acceptable fit	<0.05	<0.08	>0.9 (STD) >0.8 (GOOD)		<5	>0.9 (STD) >0.8 (GOOD)	>0.9 (STD) >0.8 (GOOD)
Obtained fit MM	0.001	0.052	0.896	269.6	1.577	0.928	0.911
Obtained fit SM	0.001	0.051	0.896	270	1.577	0.928	0.911

**Table 9:** Hypothesis testing.

Constructs	Code	Hypothesis	Relationship	β Value	Status
**Perceived Mobility**	PM	H1	PM→PU	0.511 ***	Supported
		H1a	PM→PEOU	0.582 ***	Supported
**Perceived Locational Accuracy**	PLA	H2	PLA→PEOU	0.143 *	Supported
**Effort Expectancy**	EE	H3	EE→PEOU	0.439 ***	Supported
		H3a	EE→BI	−0.052	Rejected
**Perceived ease of use**	PEOU	H4	PEOU→PU	0.531 ***	Supported
		H4a	PEOU→BI	0.371***	Supported
**Perceived Usefulness**	PU	H5	PU→BI	0.234 **	Supported
**Perceived Price**	PP	H6	PP→BI	0.210 **	Supported

***: *p* < 0.001, ** mean accepted a 0.0–2 level, *: *p* < 0.05.

## Data Availability

The data presented in this study will be available on request from the corresponding author.

## Appendix A

**Perceived Usefulness**

1. Using E-Hailing applications would enable me to access the taxi more quickly.
2. Using E-Hailing applications would make it easier to search for a taxi.
3. Using E-Hailing applications will enhance my effectiveness in searching taxis.
4. I would find E-Hailing applications useful in my daily life.
5. E-Hailing applications have improved my productivity.

**Perceived Ease of Use**

1. Learning to use E-Hailing applications would be easy for me.
2. I would find it easy to get E-Hailing applications to do what I want to do.
3. It is easy to become skillful at using e- hailing applications.
4. I would find e-hailing applications to be flexible to interact with.

**Perceived Mobility Value**

1. It is convenient to access E-Hailing anywhere at any time.
2. Mobility and E-hailing applications make it possible to get access to taxi services.
3. Mobility is an outstanding advantage of E-Hailing applications.
4. It would not require me a lot of mental effort to learn because I am skilled at mobile device functions.

**Perceived locational accuracy**

1. E-Hailing applications you are using always display accurate locations in their service.
2. E-Hailing applications always display an accurate location in real-time.
3. E-Hailing applications provide efficient routes and accurate destinations for where I want to go.
4. It provides alternative routes in rush hours

**Perceived Price**

1. E-Hailing applications are providing a low price for the customers.
2. Before requesting the pickups, the E-Hailing application revealed a higher price.
3. E-Hailing applications are not charging extra fares for any reason.
4. I believe that I can save money by using E-Hailing applications.

**Effort Expectancy**

1. Various functions of e-hailing applications are easy to locate and use.
2. The E-hailing application’s interface is clear and easy to understand.
3. The content and organization of the applications are clear and easy to understand.
4. It does not require a lot of time and effort to learn how to use e-hailing applications.

**Behavior Intention**

1. I intend to continue using E-Hailing applications during my study period.
2. I plan to continue using E-Hailing applications frequently.
3. I predict that I will use E-Hailing applications as long as I have access to it.
